# Transcriptome sequencing reveals that LPS-triggered transcriptional responses in established microglia BV2 cell lines are poorly representative of primary microglia

**DOI:** 10.1186/s12974-016-0644-1

**Published:** 2016-07-11

**Authors:** Amitabh Das, Sun Hwa Kim, Sarder Arifuzzaman, Taeho Yoon, Jin Choul Chai, Young Seek Lee, Kyoung Sun Park, Kyoung Hwa Jung, Young Gyu Chai

**Affiliations:** Institute of Natural Science and Technology, Hanyang University, Ansan, 15588 Republic of Korea; Department of Molecular and Life Sciences, Hanyang University, Ansan, 15588 Republic of Korea; Department of Bionanotechnology, Hanyang University, Seoul, 04673 Republic of Korea

**Keywords:** Gene regulation, Innate immunity, Transcription factors, Microglia, RNA sequencing

## Abstract

**Background:**

Microglia are resident myeloid cells in the CNS that are activated by infection, neuronal injury, and inflammation. Established BV2 microglial cell lines have been the primary in vitro models used to study neuroinflammation for more than a decade because they reduce the requirement of continuously maintaining cell preparations and animal experimentation models. However, doubt has recently been raised regarding the value of BV2 cell lines as a model system.

**Methods:**

We used triplicate RNA sequencing (RNA-seq) to investigate the molecular signature of primary and BV2 microglial cell lines using two transcriptomic techniques: global transcriptomic biological triplicate RNA-seq and quantitative real-time PCR. We analyzed differentially expressed genes (DEGs) to identify transcription factor (TF) motifs (−950 to +50 bp of the 5′ upstream promoters) and epigenetic mechanisms.

**Results:**

Sequencing assessment and quality evaluation revealed that primary microglia have a distinct transcriptomic signature and express a unique cluster of transcripts in response to lipopolysaccharide. This microglial signature was not observed in BV2 microglial cell lines. Importantly, we observed that previously unidentified TFs (i.e., IRF2, IRF5, IRF8, STAT1, STAT2, and STAT5A) and the epigenetic regulators KDM1A, NSD3, and SETDB2 were significantly and selectively expressed in primary microglia (PM). Although transcriptomic alterations known to occur in BV2 microglial cell lines were identified in PM, we also observed several novel transcriptomic alterations in PM that are not frequently observed in BV2 microglial cell lines.

**Conclusions:**

Collectively, these unprecedented findings demonstrate that established BV2 microglial cell lines are probably a poor representation of PM, and we establish a resource for future studies of neuroinflammation.

**Electronic supplementary material:**

The online version of this article (doi:10.1186/s12974-016-0644-1) contains supplementary material, which is available to authorized users.

## Background

It has become increasingly evident that neuroinflammation, triggered by the activation of glial cells, plays a key role in many neurodegenerative disorders, such as Alzheimer’s, Parkinson’s, and Huntington’s disease and multiple sclerosis [1, 2]. Microglia, the principal resident macrophages of the brain and spinal cord, comprise 5–12 % of brain cells and act as primary effector cells. These cells play an important role in the brain’s innate immunity, neuronal homeostasis, and neuroinflammatory pathologies [2, 3]. Microglia become rapidly activated in response to infection, inflammation, or brain injury. The activated microglia release various inflammatory mediators, including tumor necrosis factor-alpha (TNF-α), interleukin (IL)1B, IL6, nitric oxide (NO), reactive oxygen species (ROS), and prostaglandin E2 (PGE_2_) which have been implicated in various neurodegenerative diseases [1, 4]. However, the mechanisms that regulate microglial activation have not been completely defined.

Microglia express a broad range of pattern recognition receptors in the toll-like receptor (TLR) family to detect microbial intruders and brain damage [5]. Among these, bacterial cell wall endotoxin lipopolysaccharide (LPS), the ligand for toll-like receptor 4 (TLR4), is one of the most potent stimuli that induces microglial activation. LPS activates microglia, leading to the release of cytokines and a host of neurotoxic factors that induce neuronal death [6, 7]. Most research on microglial TLR signaling has been performed in vitro, usually by using cell lines, such as N9 [8] and BV2 cell lines [9]. During the past decade, in our monitoring of the PubMed search “BV2 and neuroinflammation,” we have witnessed an explosion of work aimed at understanding the role of microglia in neurodegenerative disorders. Doubt has been raised regarding the value of the BV2 cell lines as a model system. BV2 cell lines were originally derived from v-raf/v-myc-immortalized murine neonatal microglia, and this cell line is the most frequently used alternative to using primary microglia (PM). For example, previous studies reported that in the presence of LPS, transcriptomic and proteomic analyses of BV2 cell lines revealed similarities with PM [10]. Recently, other laboratories have reported that BV2 cell lines exhibit many similarities with PM and in vivo models in studies of Huntington’s disease [11]. In addition, BV2 cell lines are used in the pharmaceutical industry [12]. However, immortalization causes these cells to be different from PM in culture or in the brain [13]. For instance, previous studies have demonstrated that after exposure to macrophage colony-stimulating factor (MCSF) and transforming growth factor beta 1 (TGF-ß1), adult PM showed a unique molecular expression profile that was different from the profile in BV2 cell lines [14]. Furthermore, immortalization through transfection with oncogenes renders these cells some ways different from PM, in terms of morphology, proliferation, and adhesion [13, 15]. Thus, it remains unclear how closely established cell lines resemble PM in a comprehensive characterization of phenotypic activation.

Although a few studies have compared the effects of LPS in vitro and in vivo [10, 16], a comprehensive and comparative transcriptional profile of responses to this stimulus has not been performed using the RNA sequencing (RNA-seq) to compare results between established cell lines and PM. To analyze the transcriptomes of tissues and cells, several approaches have already been developed. Among the various available technologies, microarrays are very useful, but they provide only a semi-quantitative assessment of the transcriptome. In contrast, RNA-seq platforms are quantitative, and they provide unbiased profiles, a snapshot of the transcriptome of cells at a specific time point, and the ability to identify novel transcribed regions, unlike microarrays, and they can therefore be extremely accurate if a sufficient level of coverage is obtained [17, 18]. Using this approach, we analyzed the unbiased quantitative transcriptome of PM and compared it to that of cell lines. The outcome of these studies allowed us to identify a common and unique PM transcriptional signature distinct from the BV2 cell lines. To the best of our knowledge, our dataset is the first quantitative transcriptomic analysis to compare BV2 cell lines and PM.

## Methods

### Cell culture, stimulation, and morphological analysis of BV2 cell lines and PM

Mouse microglial BV2 cell lines were grown in high-glucose Dulbecco’s modified Eagle’s medium (DMEM) supplemented with 10 % fetal bovine serum (FBS) (catalog # 26140; Gibco, Waltham, MA), 100 IU/ml penicillin, and 10 μg/ml streptomycin (catalog # 15140; Invitrogen, Waltham, MA). The cells were maintained in a humidified incubator with 95 % air and a 5 % CO_2_ atmosphere at 37 °C. Medium containing the appropriate agents was replaced every other day. PM were isolated from 3-day-old ICR mice as previously described [19] with minor modifications. All experimental protocols were performed in accordance with the Institutional Animal Care and Use Committee (IACUC) guidelines and approved by the IACUC committee of Hanyang University (HY-IACUC-2014-0164A and HY-IACUC-2015-0075). Briefly, whole brains of neonatal mice were dissected out of the skull, and blood vessels and meninges were carefully removed. Then, the tissues from whole brains obtained from 12 mice were pooled together, finely minced, and digested using a Neural Tissue Dissociation Kit-Postnatal Neurons (Miltenyi Biotec, Germany, 130-094-802). Next, the digested cells were passed through a 70-μm nylon cell strainer (BD Bioscience, Franklin Lakes, NJ) and seeded in poly-l-lysine-coated T-75 flasks in DMEM/nutrient mixture F-12 (DMEM/F12, 1:1) containing 20 % FBS (catalog # 26140; Gibco, Waltham, MA), 100 IU/ml penicillin, and 10 μg/ml streptomycin (catalog # 15140) obtained from Invitrogen (Waltham, MA). The cells were maintained in a humidified incubator with a 95 % air/5 % CO_2_ atmosphere at 37 °C. The medium was changed every 2–3 days. After 2 weeks in culture, the mixed glial cell cultures were shaken at 150 rpm at 37 °C for 45 min, and the glial cell suspensions were collected from each flask and seeded on poly-l-lysine-coated cell culture plates. Microglial cells were sub-plated and used for further experiments. The morphology of BV2 cell lines and PM at 4 h with and without (control) treatment with LPS was analyzed for each independent experiment (Additional file 1: Figure S1A). More than 95 and 92 % of cells obtained were BV2 cell lines and PM microglia, respectively, as quantified by CD11b (rat monoclonal immunoglobulin G2b (IgG2b), clone: M1/70.15.11.5, Miltenyi Biotec Germany) FACS analysis (Additional file 1: Figure S1B). The cells were treated with LPS (10 ng/ml) and incubated for 2 and 4 h under BV2 cell lines and PM culture conditions, respectively. LPS (L6529; strain 055:B5) was purchased from Sigma-Aldrich, St. Louis, MO.

### Total RNA isolation and cDNA library preparation for transcriptome sequencing (RNA-seq)

Total RNA was extracted using RNAiso Plus (Takara Bio Inc., Shiga, Japan) and a QIAGEN RNeasy® Mini kit (QIAGEN, Hilden, Germany). BV2 cell lines or PM cells were completely lysed using RNAiso Plus, and then, 200 μl of chloroform was added. The tubes were then inverted for 5 min. The mixture was centrifuged at 12,000×*g* for 15 min at 4 °C, and the upper phase was placed into a new tube. A 600 μl volume of 70 % ethanol was added, and the mixture was applied to an RNeasy mini column. The column was washed with wash buffer. To elute the RNA, RNase-free water (30 μl) was added directly onto the RNase mini column, which was then centrifuged at 12,000×*g* for 3 min at 4 °C. To deplete ribosomal RNA (rRNA) from the total RNA preparations, a RiboMinus Eukaryote kit (Life Technologies, Carlsbad, CA) was used according to the manufacturer’s instructions. RNA libraries were created using a NEBNext® Ultra™ directional RNA library preparation kit for Illumina® (New England BioLabs, Ipswich, MA). The obtained rRNA-depleted total RNA was fragmented into small pieces using divalent cations at elevated temperatures. First-strand complementary DNA (cDNA) was synthesized using reverse transcriptase and random primers, and second-strand cDNA synthesis was then performed using DNA polymerase I and RNase H. The cDNA fragments were processed using an end-repair reaction after the addition of a single “A” base, followed by adapter ligation. These products were purified and amplified using PCR to generate the final cDNA library. The cDNA fragments were sequenced using an Illumina HiSeq2000. Biological triplicate RNA sequencing was performed on 18 independent RNA samples of BV2 cell lines and PM cells, i.e., control BV2 (3 samples), BV2 LPS 2 h (3 samples), BV2 LPS 4 h (3 samples), control PM (3 samples), PM LPS 2 h (3 samples), and PM LPS 4 h (3 samples). We selected the 2- and 4-h time point for whole-genome transcriptional profiling based on previous PCR array data that showed that the optimal induction of immune response genes occurs at this time point when microglia are activated using LPS [16, 20, 21].

### Differentially expressed gene analysis using RNA-seq data

FASTQ files from RNA-seq experiments were clipped and trimmed of adapters, and the low-quality reads were removed by the Trimmomatic [22]. Quality-controlled FASTQ files were aligned to *mus musculus* UCSC mm10 reference genome sequence using the STAR (version 2.5.1) aligner software [23] with three mismatches. To measure differential gene expression, DESeq2 [24] with the default parameters was used. A subset of condition-specific expression was defined as showing a log_2_ fold change ≥1.5 and *P* ≤ 0.01 in expression between controls and LPS-treated samples. To further characterize the BV2 cell lines and PM cells, we selected different immunoregulatory (cytokines, chemokines, interferon response genes, etc.) genes based on functionally related according to current knowledge. The RNA-seq experiments were visualized using HOMER (version 4.7) [25] after custom tracks were prepared for the UCSC Genome Browser (http://genome.ucsc.edu/). The acquired data were deposited in the Gene Expression Omnibus database under dataset accession nos. GSE79898 and GSE80304.

### Quantitative real-time RT-PCR

The reverse transcription of the RNA samples was performed as previously described [26] using 2 μg of total RNA, 1 μl of oligo (dT) primer (per reaction), and a PrimeScript 1st strand cDNA Synthesis Kit (Takara Bio Inc., Shiga, Japan). The oligo (dT) primer and RNA templates were mixed and denatured at 65 °C for 5 min and then cooled for 2 min on ice. PrimeScript buffer (5×), RTase, and RNase inhibitor were added to the cooled template mixture and incubated for 1 h at 50 °C before an enzyme inactivation step was performed at 70 °C for 15 min. Quantitative real-time RT-PCR (qRT-PCR) was performed using SYBR Green PCR Master Mix (Takara Bio Inc., Shiga, Japan) and a 7500 Fast Real-Time PCR System (Applied Biosystems, Waltham, MA). Glyceraldehyde-3-phosphate dehydrogenase (GAPDH) was used as an internal control. Complementary DNA samples were diluted 1.5-fold, and qRT-PCT was performed using an ABI-7500 Real-Time PCR System (Applied Biosystems, Waltham, MA) with SYBR Premix Ex-Taq II (Takara Bio Inc., Shiga, Japan) according to the manufacturer’s instructions. The reactions were performed in a total volume of 20 μl that contained 0.4 mM of each primer (Table 1). Each PCR series included a no-template control that contained water instead of cDNA and a reverse transcriptase-negative control for each gene. Triplicate measurements were performed for all reactions. Different samples were evaluated using 96-well plates in the gene expression experiments, and all samples were analyzed on a single plate for the endogenous control experiments. The results were analyzed using the critical threshold (∆*C*_T_) and comparative critical threshold (∆∆*C*_T_) methods in the ABI-7500 software program with the Norm finder and geNorm-plus algorithms. The primers were designed using Primer Express software (Applied Biosystems, Waltham, MA).

**Table 1:** List of primers used in qRT-PCR studies

Gene designation	Forward (5′ → 3′)	Reverse (5′ → 3′)
*CCL4*	TTCCTGCTGTTTCTCTTACACCT	CTGTCTGCCTCTTTTGGTCAG
*CCL5*	TTTGCCTACCTCTCCCTCG	CGACTGCAAGATTGGAGCACT
*CCL8*	CTGGGCCAGATAAGGCTCC	CATGGGGCACTGGATATTGTT
*CXCL1*	ACTGCACCCAAACCGAAGTC	TGGGGACACCTTTTAGCATCTT
*IFNB1*	AGCTCCAAGAAAGGACGAACA	GCCCTGTAGGTGAGGTTGAT
*IFIT1*	GCCTATCGCCAAGATTTAGATGA	TTCTGGATTTAACCGGACAGC
*IFIT2*	GGAGAGCAATCTGCGACAG	GCTGCCTCATTTAGACCTCTG
*IFIT3*	CCTACATAAAGCACCTAGATGGC	ATGTGATAGTAGATCCAGGCGT
*IRF1*	ATG CCA ATC ACT CGA ATG CG	TTG TAT CGG CCT GTG TGA ATG
*IRF2*	AATTCCAATACGATACCAGGGCT	GAGCGGAGCATCCTTTTCCA
*STAT1*	TCACAGTGGTTCGAGCTTCAG	CGAGACATCATAGGCAGCGTG
*STAT2*	GTTACACCAGGTCTACTCACAGA	TGGTCTTCAATCCAGGTAGCC
*KDM4A*	GAC CAC ACT CTG CCC ACA C	TCC TGG GGT ATT TCC AGA CA
*SETDB2*	TGGGTCTGCCACAAATGGAG	TCCAGTGTTTGCGTGTTACTC
*GAPDH*	TGCGACTTCAACAGCAACTC	CTTGCTCAGTGTCCTTGCTG

### Functional annotation

To functionally annotate the most significant genes, gene ontology analysis was performed using DAVID (Database for Annotation, Visualization and Integrated Discovery), version 6.8 [27]. Gene ontology was analyzed using a modified Fisher’s exact *P* value in the DAVID program. *P* values less than 0.001 were considered to be greatly enriched in the annotation category.

### Canonical pathway analysis of datasets

An Ingenuity Pathway Analysis (IPA) (Ingenuity Systems, http://www.ingenuity.com, CA) was performed to analyze the most significant canonical pathways in the datasets as previously described [28]. The genes from datasets associated with canonical pathways in the Ingenuity Pathways Knowledge Base (IPAKB) were considered for literary analysis. The significance of the associations between datasets and canonical pathways was measured in the following manner: (1) the ratio of the number of genes from the dataset that mapped to a canonical pathway was divided by the total number of genes that mapped to the same canonical pathway and (2) Fisher’s exact test for a *P* value indicating the probability that the association could be explained by chance. After uploading the datasets, gene identifiers were mapped to corresponding gene objects, and the genes were overlaid onto a global molecular network in the IPAKB. Gene networks were algorithmically generated based on connectivity.

### Transcription factor-binding motif enrichment analysis

NCBI reference sequence messenger RNA (mRNA) accession numbers were subjected to transcription factor-binding motif analysis using the web-based software Pscan [29]. The JASPAR [30] database of transcription factor (TF)-binding sequences was analyzed using enriched groups of −950 base pair (bp) sequences to +50 bp of the 5′ upstream promoters. The range −950 to +50 was selected from the range options in Pscan to obtain the best coverage for a −1000- to +50-bp range.

### Statistical analysis

The data were analyzed using Origin Pro 8 software (Origin Lab Corporation, Northampton, MA). Each value is expressed as the mean ± standard error of the mean (SEM). All qRT-PCR data were analyzed with SPSS 17.0 software (SPSS Inc., Chicago, IL). The data were tested using one-way ANOVA followed by Tukey’s HSD post hoc test. *P < 0.01 and **P < 0.001 were considered significant.

## Results

### Gene-induction patterns following TLR4 activation in PM

**Fig. 1:**
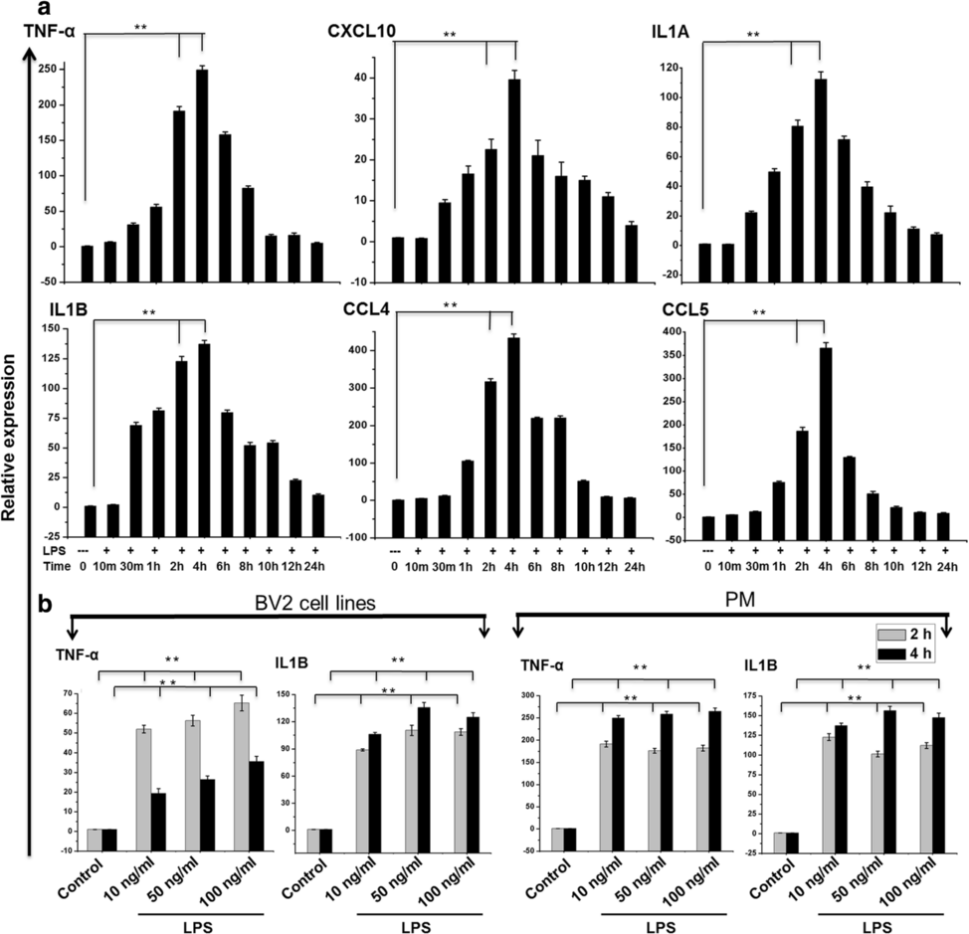
Induction of inflammatory response-related genes following TLR4 activation. **a** Quantitative real-time reverse transcriptase-PCR analysis of the expression of inflammatory genes in PM stimulated with LPS (10 ng/ml). The expression of inflammatory genes was significantly up-regulated at the indicated times in cells treated with LPS (10 ng/ml) compared to untreated cells. **b** BV2 cell lines or PM were stimulated with different doses of LPS (10–100 ng/ml) for 2 and 4 h before analysis of inflammatory response-related genes by quantitative real-time reverse transcriptase-PCR analysis. Gene expression was normalized to GAPDH transcript levels. The data represent three biologically independent experiments. The values are the mean ± SD of triplicate wells. *P < 0.01 and **P < 0.001 compared to the control

To determine the proper time course responses, we performed an expression analysis in TLR4-stimulated versus control PM. LPS (10 ng/ml) caused a transient up-regulation of key inflammatory response-related genes, peaking at 2 and 4 h for TNF-α, CXCL10, IL1A, IL1B, CCL4, and CCL5 (Fig. 1a). In response to stimulation by different doses of LPS (10–100 ng/ml), BV2 cell lines and PM caused significant up-regulation of key inflammatory response-related genes at 2 and 4 h. The fold induction in the increase of TNF-α and IL1B in response to different doses of LPS (10–100 ng/ml) was similar in both cell types at 2 and 4 h (Fig. 1b). We hence used the lower doses for subsequent analyses. Notably, morphological analysis shows that at 4 h, LPS-treated BV2 cell lines and PM show similar morphology and responses as compared to control cells (Additional file 1: Figure S1A).

### RNA-seq transcriptional comparison between PM and BV2 cell line microglia following TLR4 activation

**Fig. 2:**
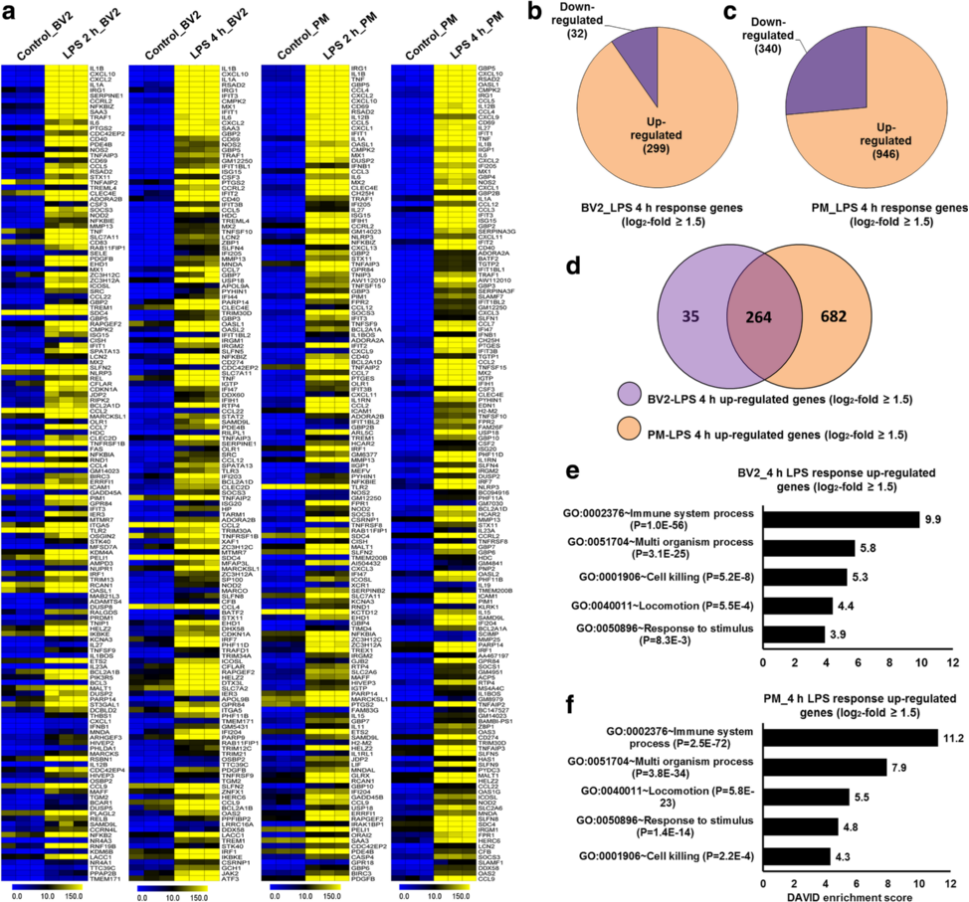
RNA-seq analyses reveals LPS-induced inflammatory response-related genes and their downstream effectors in BV2 cell lines and PM. **a** A heat map representing the top 150 inflammatory genes that were up-regulated by 2- and 4-h LPS stimulation in BV2 cell lines and PM (*P* ≤ 0.01, and log_2_ fold change ≥1.5). Each *row* shows the relative expression level for a single gene, and each *column* shows the expression level of a single sample. Biological replicates (*n* = 3) for each condition were performed. **b**, **c** Pie chart displaying the number of up or down-regulated genes at 4-h LPS stimulation in BV2 cell lines and PM. **d** The *area of overlap* indicates the number of unique or shared up-regulated genes after 4 h of LPS stimulation in BV2 cell lines and PM. **e**, **f** Gene ontology analysis of the functional annotations that were associated with the top 150 up-regulated genes at 4 h after LPS stimulation in the BV2 cell lines and PM

To gain a comprehensive understanding of the mechanisms involved and to directly compare how TLR4 stimulation alters the transcriptomic profile of BV2 cell lines and PM, RNA-seq experiments were performed. Next, we performed principal component analysis (PCA) using DESeq2 to examine congruency among biological replicates. PCA analysis showed a good separation and high level of consistency between biological replicates of the same population in BV2 cell lines and PM (Additional file 2: Figure S2). According to the above criteria, 237 genes for 2 h and 331 genes for 4 h were differentially regulated following LPS treatment BV2 cell lines. Of these, 205 and 299 genes were up-regulated, and 32 genes were down-regulated at 2 and 4 h, respectively, after LPS treatment in BV2 cell lines (Fig. 2b; Additional file 3: Figure S3). Surprisingly, we found significant generational differences in PM, in which 531 genes for 2 h and 1286 genes for 4 h were differentially regulated. Of these, 362 and 946 genes were up-regulated, and 169 and 340 genes were down-regulated at 2 and 4 h, respectively, after LPS treatment (Fig. 2c; Additional file 3: Figure S3), in contrast to our previous studies showing that differentially expressed genes in the BV2 cell lines were less pronounced at 2 and 4 h after LPS treatment [20, 31]. In this analysis, we have increased biological duplicate to biological triplicate RNA-seq as well as *P* value less than 0.01 for differential expression genes, which reduces the number of differential expression genes in the BV2 cell lines after 2- and 4-h LPS treatment. In addition, differences in the passage number and intrinsic variability of cells may lead to these discrepancies. The genes were grouped into several categories based on their biological processes and molecular gene ontology functions, and heat maps were generated to aid the visualization of the gene expression pattern. The top 150 inflammatory genes that were up-regulated at 2 and 4 h after LPS stimulation in BV2 and PM cells are shown in Fig. 2a. Next, we performed functional classification analyses of the up-regulated genes using DAVID Bioinformatics Resources [27] by classifying the results into gene ontology (GO) categories (FDR 0.05) using the biological process (BP) and molecular function (MF) categories. We observed that in both BV2 cell lines and PM, the top 150 genes that were up-regulated in response to 4-h LPS stimulation were mainly involved in the immune system process and multi-organism processes (Fig. 2e, f). Because the down-regulated genes were not associated with inflammation, only the up-regulated genes were further studied. We confirmed using GO analysis (FDR 0.05) and DAVID Bioinformatics Resources that the top 150 transcripts that were down-regulated by 4-h LPS stimulation in PM were associated with the developmental process and regulation of GTPase activity (Additional file 4: Figure S4).

### Common and unique characteristics of PM versus BV2 cell lines

To further investigate common and unique characteristics between LPS-treated PM or the BV2 cell lines, we again used RNA-seq data to compare the transcriptome of BV2 cell lines with that of PM. In a similar approach (see the “Methods” section), we compared the transcripts in LPS-treated BV2 cell lines with those of PM. Differential expression analysis clearly revealed that LPS elicited the induction of a unique gene set in response to stimulation with this TLR ligand at the 2- and 4-h time point in BV2 cell lines and PM cells (Fig. 2d; Additional file 3: Figure S3) suggesting a substantial number of dissimilarities between the two cell types. PM cells up-regulated 220 for 2 h and 682 genes for 4 h that are not common to the BV2 cell lines. In contrast, BV2 cell lines up-regulated 63 for 2 h and 35 genes for 4 h that are not common to the PM cells (Fig. 2d; Additional file 3: Figure S3). The unique gene set is presented at the 2- and 4-h time point in BV2 cell lines in Additional file 5: Table S1. However, PM and the BV2 cell lines also had similarities in their transcriptomes. Of the up-regulated genes, BV2 cell lines and PM shared 142 genes for 2 h and 264 genes for 4 h following LPS treatment (Fig. 2d; Additional file 3: Figure S3). Importantly, this technology allowed us to identify several specific gene families involved in immune responses that were uniquely altered in LPS-treated PM cells. We found that LPS elicited the induction of unique 10 cytokines, 9 chemokines, 13 (interferon (IFN))-regulated genes (IRGs), 9 TFs, 3 epigenetic regulators, and 11 undetected transcripts in response to stimulation with this TLR ligand at the 4-h time point in PM cells (Figs. 4b, 5b, and 6f). The following inflammatory response- and immune response-related genes were markedly affected only in PM: cytokines/chemokines (CCL6, CCL8, CX3CL1, CXCL1, CXCL3, CXCL9, CXCL11, CXCL16, IL12B, IL18BOS, IL18BP, IL19, IL23A, IL27, IRAK1BP1, SOCS1, TNFSF11A, and TNFSF15), IRGs (GBP2B, GBP4, GBP9, GBP10, GBP11, IFI44I, IFIH1, IFNB1, etc.), TFs (IRF2, IRF5, IRF8, STAT5A, etc.), epigenetic regulators (KDM1A, NSD3, and SETDB2), and undetected transcripts (CLEC4A1, CLEC7, CLEC7A, GPR18, MMP3, MMP9, MMP12, etc.). These data suggest that following LPS treatment, PM express a unique set of genes, distinct from that of BV2 cell lines, which may offer potential targets for further investigations into microglia biology.

### Canonical pathway prediction modulated through TLR4-stimulated PM and BV2 cell lines

**Fig. 3:**
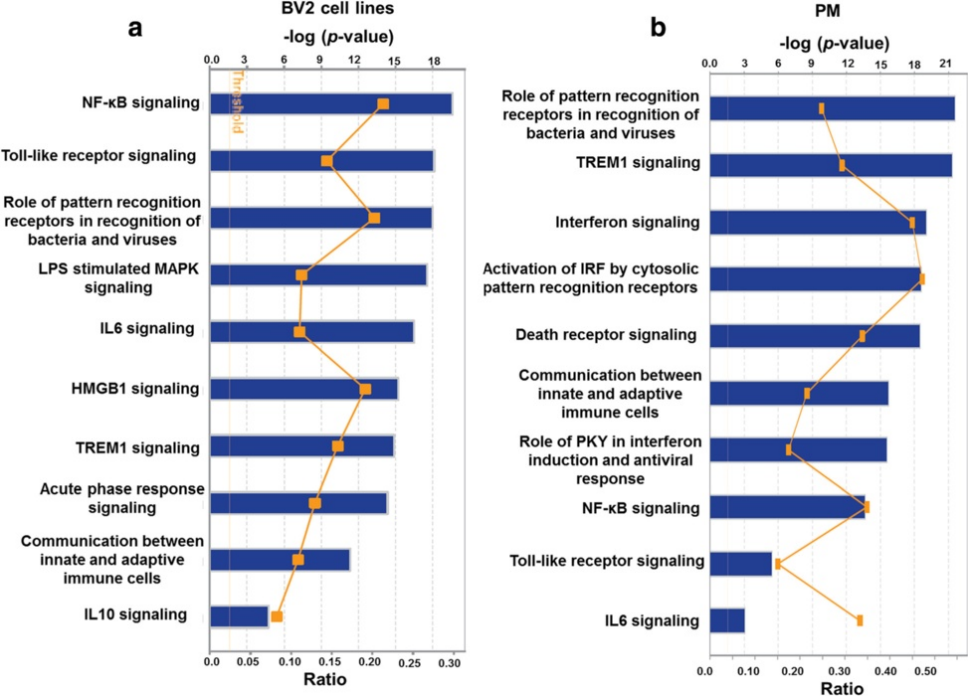
Top IPA-based canonical pathway analyses at 4 h after LPS stimulation in BV2 cell lines and PM. **a**, **b** Ingenuity® Bioinformatics pathway analysis revealed that highly canonical pathways were differentially expressed in BV2 cell lines and PM cells after LPS stimulation. The canonical pathways included in this analysis are shown along the *y*-axis of the bar chart. The *x*-axis indicates the statistical significance. Calculated using the right-tailed Fisher exact test, the *P* value indicates which biologic annotations are significantly associated with the input molecules relative to all functionally characterized mammalian molecules and the *yellow threshold line* represents the default significance

To gain further understanding into the molecular functions of up-regulated genes, we performed IPA (IPA, Ingenuity Systems, http://www.ingenuity.com) [28] to identify the canonical pathways that represent the relevant molecular functions based on functional knowledge inputs. IPA analysis of transcriptome profiling data revealed the highly statistically significant regulation of well-known TLR4-mediated pathways that are important in immune responses, including genes involved in the roles of pattern recognition receptors during the recognition of bacteria and viruses, interferons, and NF-kB signaling, in BV2 cell lines and PM (Fig. 3a, b). Other notable pathways included the TREM1, toll-like receptor, and death receptor signaling pathways. The up-regulation of these functions in response to stimulation with TLR4 stimulation is interesting and indicates that our approach for comparing BV2 cell lines and PM is strong but rather predictable. Interestingly, the pathways that scored as unique in PM relative to microglial cell lines were involved in interferon signaling. The biggest difference between PM and microglial cell lines was therefore a difference in interferon signaling according to IPA analysis. This may reflect the interferon signaling that is known to occur in PM following immune activation.

### The expression of proinflammatory cytokines, chemokines, and interferon response transcripts are highly expressed in PM than BV2 cell lines following 2- and 4-h stimulation with TLR4

**Fig. 4:**
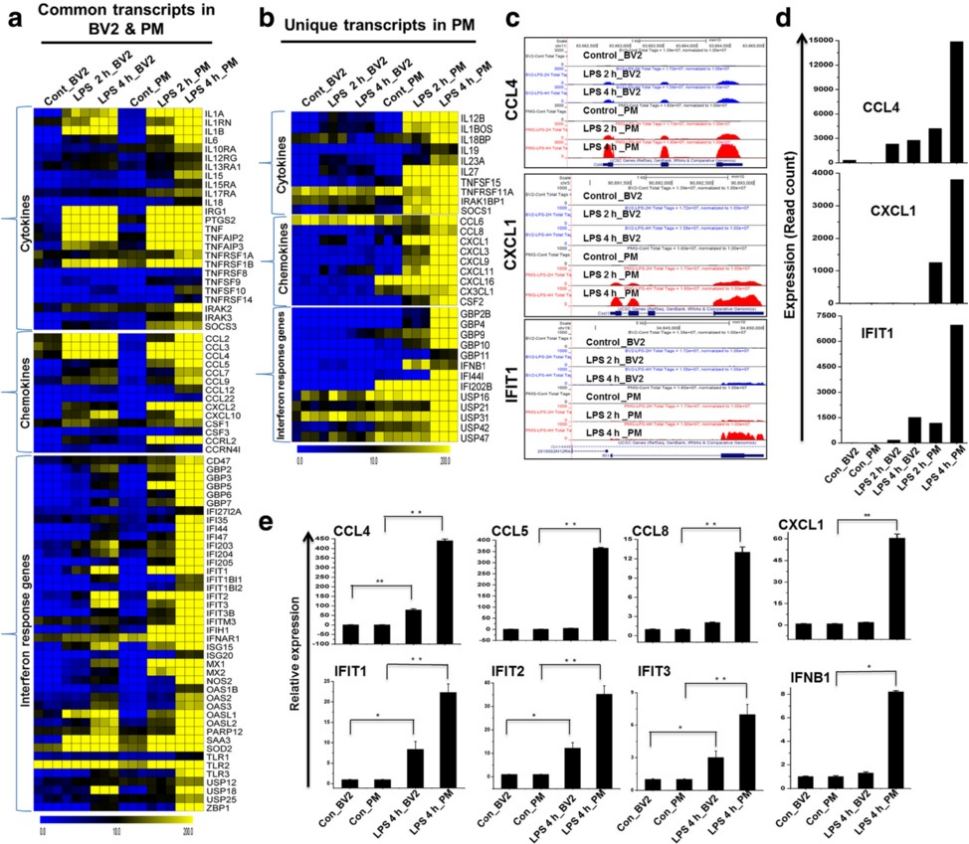
Differences in transcriptomic profiles (cytokines, chemokines, and interferon response genes) between established BV2 cell lines and PM. **a** Heat map representation depicting the common expression of positive regulators of inflammatory genes between BV2 cell lines and PM cells after 2- and 4-h LPS stimulation. **b** Heat map representation of the positive regulators of inflammatory transcripts that were unique to PM cells, which showed a distinct signature after 2- and 4-h LPS stimulation compared to BV2 cell lines. **c** UCSC Browser images representing normalized RNA-seq read densities. **d** Transcript abundance (in read count) was evaluated using RNA-seq in 2- and 4-h LPS-induced BV2 cell lines and PM cells. **e** Quantitative real-time reverse transcriptase-PCR analysis of LPS-induced positive regulators of inflammatory gene expression (cytokines, chemokines, and interferon response genes) that were common and unique to PM compared to BV2 cell lines. Gene expression was normalized to the GAPDH transcript levels. *P < 0.01 and **P < 0.001 compared to the control. The data represent three biologically independent experiments

To identify differences in the regulation of these genes in PM compared to BV2 cell lines, cells were treated with LPS to analyze change in cytokine, chemokine, and interferon response gene expression. Our RNA-seq analysis revealed that almost all of the proinflammatory cytokines/chemokines were more highly induced in PM than in the BV2 cell lines (Fig. 4a, c, d). Importantly, PM expressed several unique cytokines/chemokines that were not expressed in the BV2 cell lines (Fig. 4b). These included CCL6, CCL8, CX3CL1, CXCL1, CXCL3, CXCL9, CXCL11, CXCL16, IL12B, IL18BOS, IL18BP, IL19, IL23A, IL27, IRAK1BP1, SOCS1, TNFSF11A, and TNFSF15. Interestingly, the CXCL10-associated CXCR3-binding molecules CXCL9 and CXCL11 were highly enriched only in PM cells, while CXCL4 was not. The growth-regulated oncogenes (GRO) (CXCL1 and CXCL3) also displayed a similar trend: they were expressed only in PM. Monocyte chemoattractant proteins (MCP) 1 and 3 (CCL2 and CCL7, respectively) were more highly induced in PM than in the BV2 cell lines (Fig. 4a). However, CCR7, which is a dendritic cell antigen that is expressed in microglia in inflamed central nervous system (CNS) tissues [32], stromal-derived factor 1 (SDF-1, also CXCL12), and the recently discovered chemokine CCL25 were not induced in either PM or the BV2 cell lines. Engagement of TLR activates signaling pathways that lead to transcriptionally induce hundreds of IRGs. Type I and type II have well-described antiviral properties, and most of the IRGs are regulated by both type I and type II IFNs [33]. These IRGs included factors known to be involved in antiviral responses. To further characterize the similarities and differences between the analyzed BV2 cell lines and PM, we compared the levels of expression of IRGs. These highly induced genes included the IFN-induced protein with tetratricopeptide (IFIT) family gene IFIT1 (p56), IFIT2, IFIT3, IFIH1, IFITM3, the IRF3-dependent gene ISG15 [34], ISG20, IFI35, IFI44, IFI47, IFI203, IFI204, IFI205, OASL, OASL2, OAS3, etc., with their expression in PM being higher than their expression in the BV2 cell lines (Fig. 4a). Importantly, the PM expressed several IRGs that were not expressed in the BV2 cell lines (Fig. 4b). RNA-seq analysis revealed that 43 IRGs were significantly up-regulated in both BV2 cell lines and PM. Importantly, we found that almost all IRG BV2 cell line response was weaker than the response of PM. A better understanding of the consequences of LPS-stimulated IRG production in PM warrants a comprehensive investigation. These included GBP4, GBP6, IFNB1, and MX2 [35]. However, we did not detect other IRGs, such as IFITM1 and GBP1, in either the PM or the BV2 cell lines. These data strongly suggest that LPS induces proinflammatory cytokines, chemokines, and IRGs more strongly and broadly in PM cells than in BV2 cell lines.

### PM are hyperresponsive and express higher levels of multiple families of TFs than BV2 cell lines following 2- and 4-h stimulation with TLR4

**Fig. 5:**
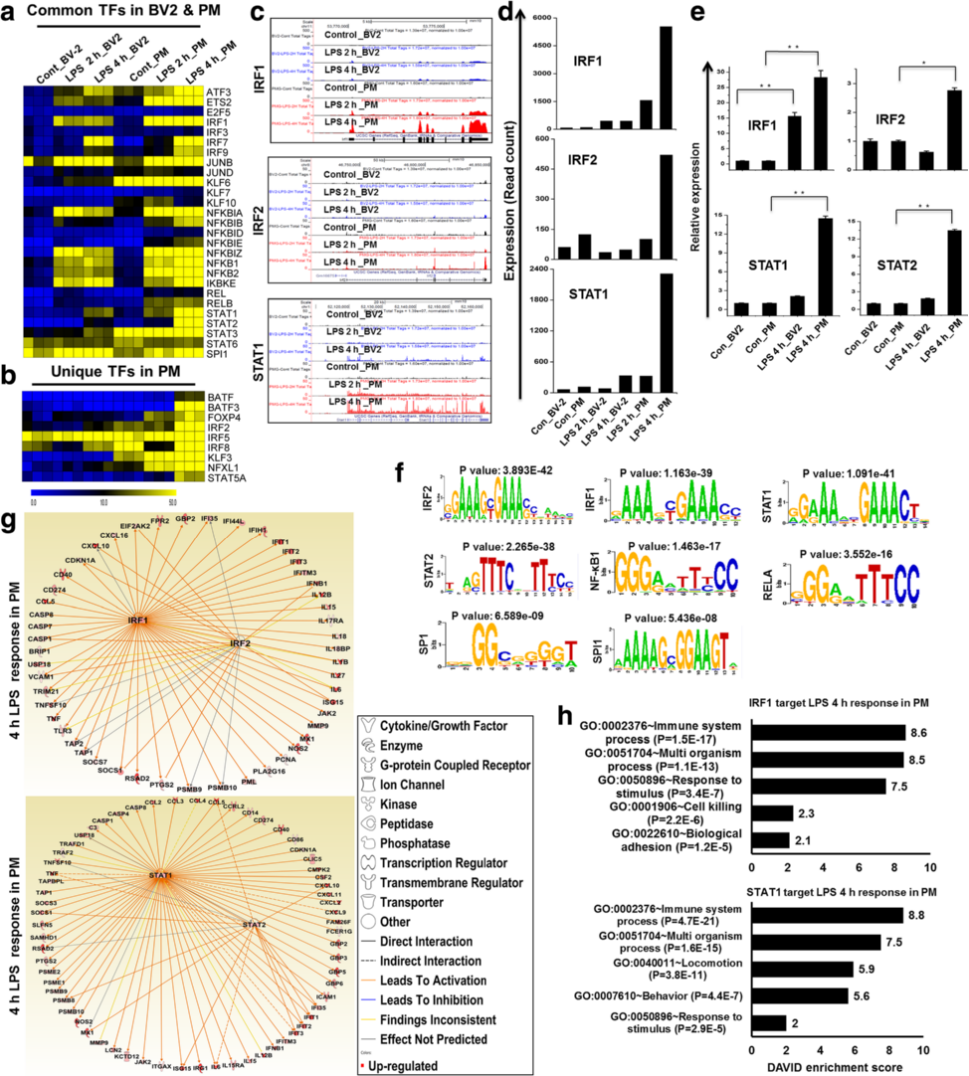
Differences in the expression of selected TF families between BV2 cell lines and PM. **a** Heat map representation showing the commonly expressed TF families between BV2 cell lines and PM cells after 2- and 4-h LPS stimulation. **b** Heat map of the TF families that were unique to PM cells, which showed a distinct signature following 2- and 4-h LPS stimulation compared to BV2 cell lines. **c** UCSC Browser images representing normalized RNA-seq read densities. **d** Transcript abundance (in read count) was evaluated using RNA-seq in 2- and 4-h LPS-induced BV2 cell lines and PM cells. **e** Confirmation of differentially expressed TFs was performed using quantitative reverse transcription-polymerase chain reaction. The genes that were common and unique to PM cells compared to BV2 cell lines are shown. Gene expression was normalized to GAPDH transcript levels. *P < 0.01 and **P < 0.001 compared to the control. The data represent three biologically independent experiments. **f** Patterns of TF motif enrichment within the promoters of the indicated genes in 4-h LPS-induced PM cells. **g** The activity of highly connected positive regulators of the inflammatory genes IRF1, IRF2, STAT1, and STAT2 led to the activation of this network, as assessed using the IPA molecule activity predictor in LPS-induced PM cells. **h** Results of the GO term analysis using DAVID. The genes that were regulated by STAT1 and IRF1 in response to LPS in PM cells are shown

To better understand the relationship between PM and BV2 cell lines, we reassessed the RNA-seq data. TFs, including IRF, Kruppel-like factor (KLF), NF-kB, and signal transducer and activator of transcription (STAT), are important in inflammatory diseases [36, 37]. The following TF families exhibited the most dramatic levels of induction following LPS challenge in PM: the NF-kB group of TFs (NF-kBIA, NF-kBIB, NF-kBID, NF-kBIE, NF-kBIZ, NF-kB1, NF-kB2, REL, and RELB), the interferon group of TFs (IRF1, IRF2, IRF5, IRF7, IRF8, and IRF9), and the STAT group of TFs (STAT1, STAT2, STAT3, and STAT5A) (Fig. 5a–d). However, other members of the IRF and STAT families were unaffected by treatment with LPS, suggesting that LPS induces TFs in a highly selective manner in microglia. More importantly, in agreement with a previous study performed in our laboratory, we observed that IRF2, IRF5, STAT1, STAT2, and STAT5A were not expressed in LPS-stimulated BV2 cell lines [20], suggesting that these TFs might be important regulators of the selective inflammatory gene expression that occurs in PM. In addition, our RNA-seq analysis revealed that these TFs were more highly induced in PM than in BV2 cell lines. Furthermore, we also found other groups of TFs (ATF3, E2F5, ETS2, and FOXP4) were highly up-regulated in PM (Fig. 5a, b).

Next, we conducted a TF motif analysis to analyze LPS-induced gene expression in BV2 cell lines and PM. We used the Pscan software tool [29] to perform an in silico computational analysis to determine the over-represented *cis*-regulatory elements within the 5′-promoter regions of coordinately regulated genes. Applying this score to the promoters of the genes that were differentially expressed in response to LPS revealed that the putative binding sites for eight TFs (IRF1, IRF2, STAT1, STAT2, NF-kB1, RELA, SP1, and SPI1) were significantly enriched in PM (Fig. 5f), suggesting that these TFs might be involved in the regulation of LPS-induced gene expression in PM. Nevertheless, further TF-targeted studies are required to validate this regulation in PM. In addition to a TF motif analysis, we also used IPA software [28] to identify the target genes that were directly or indirectly activated by the TFs that were identified to be activated in response to LPS stimulation. Importantly, we found that the expression of the majority of the cytokines and chemokines was directly regulated by the TFs that were identified as selectively enriched in PM, including IRF1, IRF2, STAT1, STAT2 (Fig. 5g and Table 2). To further functionally classify the STAT1, and IRF1-regulated genes, they were functionally annotated using the DAVID 6.8 software package [27]. Interestingly, we observed strong enrichment in the GO terms for the IRF1, and STAT1 regulated transcripts that were associated with immune system processes in the LPS-stimulated PM (Fig. 5h), suggesting that IRF1, IRF2, STAT1, and STAT2 might also be involved in the regulation of inflammation in microglia.

**Table 2:** Leads to activation of inflammatory genes by identified TFs in response to 4-h LPS stimulation in PM

STAT1 predicted to be activated (65 genes) (*P* = 1.41 E-74)	STAT2 predicted to be activated (14 genes) (*P* = 1.03 E-41)	IRF1 predicted to be activated (51 genes) (*P* = 4.98 E-61)	IRF2 predicted to be activated (21 genes) (*P* = 3.55 E-35)
CASP1, CASP4, CASP8, CCL2, CCL3, CCL4, CCL5, CCRL2, CD14, CD274, CD40, CD86, CDKN1A, CLIC5, CMPK2, CSF2, CXCL10, CXCL11, CXCL2, CXCL9, FAM26F, FCER1G, GBP2, GBP3, GBP5, GBP6, ICAM1, IFI35, IFIT1, IFIT2, IFIT3, IFITM3, IFNB1, IL12B, IL15, IL15RA, IL6, IRG1, ISG15, ITGAX, JAK2, KCTD12, LCN2, MMP9, MX1, NOS2, PSMB10, PSMB9, PSMB8, PSME1, PSME2, PTGS2, RSAD2, SAMHD1, SLFN5, SOCS1, SOCS3, TAP1, TAPBPL, TNF, TNFSF10, TRAF2, TRAFD1, USP18, C3	CCL5, CXCL10, IFI35, IFIT1, IFIT2, IFIT3, IL6, ISG15, MX1, PSMB8, RSAD2, SOCS1, TNF, TNFSF10	TRIM21, TNFSF10, TNF, TLR3, TAP2, TAP1, SOCS7, SOCS1, RSAD2, PTGS2, PSMB9, PSMB10, PML, PLA2G16, PCNA, MX1, NOS2, MMP9, JAK2, ISG15, IL6, IL27, IL1B, IL18BP, IL18, IL17RA, IL15, IL12B, IFNB1, IFITM3, IFIT3, IFIT2, IFIT1, IFIH1, IFI44L, IFI35, GBP2, FPR2, EIF2AK2, CXCL16, CXCL10, CDKN1A, CD40, CD274, CCL5, CASP8, CASP7, CASP1, BRIP1, USP18, VCAM1	USP18, VCAM1, TRIM21, TNFSF10, TLR3, TAP2, TAP1, SOCS1, PTGS2, PSMB9, PSMB10, ISG15, IL6, IL1B, IL12B, IFNB1, IFI35, EIF2AK2, CXCL10, CDKN1A, CASP1

### A comparison of epigenetic regulators and undetected transcripts between BV2 cell lines and PM following 2- and 4-h stimulation with TLR4

**Fig. 6:**
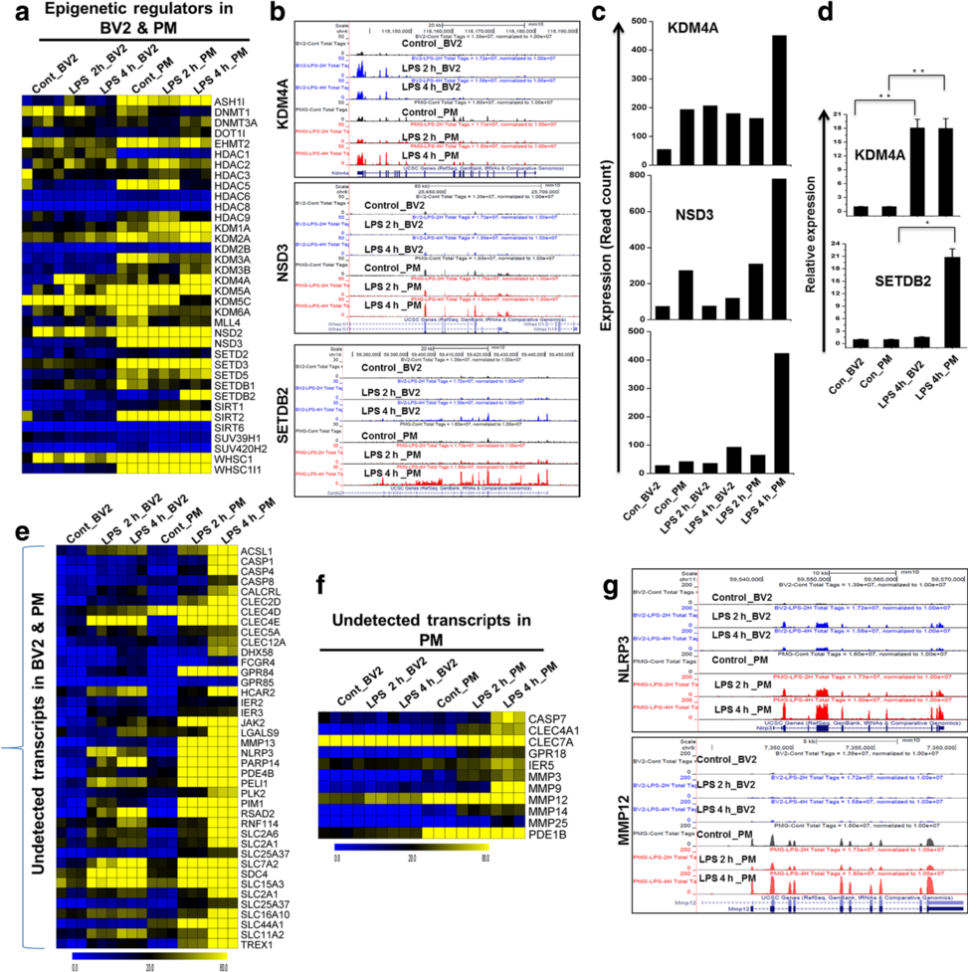
Identification of novel epigenetic regulators and inflammatory-related genes in LPS-induced PM cells. **a** Heat map representation showing the unique and common expression of epigenetic regulators in BV2 cell lines and PM cells after 2- and 4-h LPS stimulation. **b** UCSC Browser images representing normalized RNA-seq read densities. **c** Transcript abundance (in read count) was evaluated using RNA-seq in 2- and 4-h LPS-induced BV2 cell lines and PM cells. **d** Quantitative real-time reverse transcriptase-PCR analysis of the expression of epigenetic regulators showing the markers that were common or unique to PM cells and BV2 cell lines cells that were stimulated with 4-h LPS. Gene expression was normalized to GAPDH transcript levels. *P < 0.01 and **P < 0.001 compared to the control. The data represent three biologically independent experiments. **e**, **f** Heat map representation showing the common (*left panel*) and unique expression (*right panel*) profiles of novel inflammatory-related genes between BV2 cell lines and PM cells after 2- and 4-h LPS stimulation. **g** UCSC Browser images representing normalized RNA-seq read densities of novel inflammatory-related genes after 2- and 4-h LPS stimulation in BV2 cell lines and PM cells compared to the controls

To further characterize the degree of similarity or dissimilarity between BV2 cell lines and PM, we compared the most significant epigenetic regulators between these two groups. We defined this as genetic control via factors other than the DNA sequence [38]. A high level of induction of the epigenetic regulator histone methyltransferase (SETDB2) has recently been demonstrated to potentiate innate immune responses [39]. We previously showed that DNA methyltransferase (DNMT3L) and histone demethylases (KDM4A) were specifically up-regulated after LPS stimulation in microglial BV2 cell lines [20]. To our surprise, in this study, we found that in addition to DNMT3L and KDM4A, histone-lysine *N*-methyltransferase NSD3, lysine (K)-specific demethylase 1a (KDM1A), and histone methyltransferase SETDB2 were also significantly up-regulated in PM compared to BV2 cell lines, indicating that KDM1A, NSD3, and SETDB2 may be involved in the selective regulation of PM functions (Fig. 6a–c). However, we could not identify any histone deacetylase genes (HDAC) in LPS-induced BV2 cell lines and PM (Fig. 6a). The neuroinflammatory response to activation states has also been implicated to involve microRNAs (miRNAs). miRNAs are a class of small non-coding RNA molecules, ~22 nucleotides in length that function in the post-transcriptional regulation of gene expression. Dysregulation of miRNA expression has been demonstrated to play critical roles in the pathogenesis of CNS diseases. For example, during the onset of experimental autoimmune encephalomyelitis (EAE), miR-124 is down-regulated in microglia, while transfection of miR-124 leads to marked reduction in the severity of EAE, suggesting that miR-124 can be a target for neuroprotection [40]. Nevertheless, in the presence of LPS exposure, detailed miRNA profiling will be required to determine the unique miRNA signature and their selective roles to regulate inflammatory genes in microglial BV2 cell lines. This is an exciting area that we are keenly pursuing further. In addition to differentially expressed cytokines/chemokines, TFs, and epigenetic regulators, annotation of the RNA-seq data also revealed approximately 11 previously undetected genes that were specifically up-regulated in PM. Furthermore, we found that approximately 40 previously undetected genes were significantly up-regulated in PM compared to BV2 cell lines (Fig. 6e–g).

### Confirmation of differentially expressed genes by qRT-PCR

A large number of genes that were identified to be differentially regulated using RNA-seq analysis were subjected to validation using qRT-PCR. GAPDH was used as the reference gene. Most of the genes were selected for validation based on whether they were selectively altered by LPS stimulation in PM. To measure gene expression, mRNA was reverse transcribed into cDNA using PrimeScript TM Reverse Transcriptase (Takara Bio Inc., Shiga, Japan), and the qRT-PCR assays were repeated several times using at least three mRNA preparations from independent experiments. The results are expressed as the fold change relative to the control levels. Sixteen genes were selected for verification, and the RNA-seq expression patterns were confirmed for 14 of these genes (CCL4, CCL5, CCL8, CXCL1, IFIT1, IFIT2, IFIT3, IFNB1, IRF1, IRF2, STAT1, STAT2, KDM4A, and SETDB2; Figs. 4e, 5e, and 6d). Two genes (KLF7 and IRAK3) were found to be non-significantly altered (data not shown) in the qRT-PCR analysis compared to the RNA-seq experiments. Furthermore, we previously confirmed that the levels of the cytokines, chemokines, and several transcripts in the supernatants were significantly up-regulated in PM cells compared to the BV2 cell lines [31].

## Discussion

Using an RNA-seq approach, in the present study, we show that unique transcriptional changes occur in mouse PM following inflammatory stimulation that distinguished these cells from microglial BV2 cell lines. Although a few studies have attempted to use microarrays to produce comparative transcriptional profiles between BV2 cell lines and PM, such studies are limited because this technology provides only a semi-quantitative assessment of the transcriptome [10, 16]. The strength of our analysis, which was aimed at obtaining comprehensive and comparative transcriptional profiles of responses to inflammatory stimulation, was enhanced by the use of RNA-seq to analyze differences between BV2 cell lines and PM. The data obtained in our study correlate to a large extent with those demonstrated by other studies, including [10, 16]. In contrast to our findings, the above studies demonstrated that in the presence of LPS (100 ng/ml), PM cells up-regulated only 118 genes at the 4-h time point and of the up-regulated genes, BV2 cell lines shared most of the genes to PM using microarray experiments [10, 16]. The present study not only greatly extends earlier findings but also distinguishes between BV2 cell lines and PM in response to LPS. These results allowed us to discover novel transcriptional alterations that occurred in PM but were not detected in BV2 cell lines. The surprising and unexpected finding of our study was that PM reacted stronger to LPS and that therefore, a much larger number of transcripts, including many novel transcripts, were altered in PM than in BV2 cell lines. Therefore, there is a potentially enormous qualitative difference in responses to inflammatory stimulus between PM and BV2 cell lines. Importantly, Cao et al. [41] reported that upon second-hit LPS exposure, heme oxygenase 1 (HMOX1) and fructose-1,6-bisphosphatase (FBP) genes as well as HDAC 1, 2, 4, and 6 were uniquely differentially expressed in microglia, which have potential role for desensitization. In our RNA-seq data, we could not identify any histone deacetylases as well as FBP genes in LPS-induced BV2 cell lines and PM. However, we observed HMOX1 was significantly expressed in both BV2 cell lines and PM in response to 4-h LPS (Additional file 6: Figure S5). Nevertheless, whether this gene has any functional role on LPS-mediated modulation of microglia activation will require further study.

These results showed that cytokines/chemokines, antiviral genes, and IRGs that are associated with inflammation were significantly up-regulated in response to LPS and that these changes were stronger in PM than in BV2 cell lines microglia (Fig. 4a–e). Both the number of genes and the extent of the fold changes in the commonly altered genes were significantly more modulated in PM compared to BV2 cell line microglia. PM cells up-regulated 220 for 2 h and 682 genes for 4 h that are not common to the BV2 cell lines (Fig. 2d; Additional file 3: Figure S3). Importantly, our RNA-seq analysis is the first to identify several important differences in the expression patterns of cytokines/chemokines, antiviral genes, and IRGs that were not previously identified to be activated by LPS in BV2 cell lines but that were found to be altered in PM (Fig. 4b). In particular, this technology allowed us to identify 10 cytokines, 9 chemokines, and 13 IRG genes that were uniquely altered in LPS-stimulated PM cells. The following inflammatory response- and immune response-related genes were markedly affected only in PM: cytokines/chemokines (CCL6, CCL8, CX3CL1, CXCL1, CXCL3, CXCL9, CXCL11, CXCL16, IL12B, IL18BOS, IL18BP, IL19, IL23A, IL27 IRAK1BP1, SOCS1, TNFSF11A, and TNFSF15) and IRGs (GBP2B, GBP4, GBP9, GBP10, GBP11, IFI44I, IFIH1, IFNB1, etc.).

Cytokines and chemokines are involved in the regulation of inflammation, and the excessive production of these molecules has been associated with disease progression, severe neuroinflammation pathologies, and synaptic transmission [42]. IL12 plays an important role in early inflammatory responses to infection, and increased IL12 expression can be dangerous to the host because it is involved in the pathogenesis of a number of autoimmune inflammatory diseases, including multiple sclerosis (MS) [43]. TNFSF15 is one of the more recently identified TNF ligands. TNFSF15 is a multifunctional, specialized cytokine that is involved in the modulation of inflammation [44]. The chemokine CCL5 has been implicated in a wide array of pathological conditions in the brain and in neurodegenerative diseases. In particular, abnormal CCL5 expression was observed in the cerebrospinal fluid (CSF) of patients suffering from MS and in the CNS in mice with EAE [45]. In addition, Si et al. [46] reported elevated levels of CCL5 in HIV-1 viral protein R-induced human microglial cells. Importantly, BV2 cell lines exposed to these factors did not or less induce the expression of these inflammatory response-related genes than PM.

We observed that LPS significantly induced the expression of several genes known to be involved in antiviral immunity, and we found that these changes in signaling were more potent in PM than in BV2 cell lines (Fig. 4a, b). Unexpectedly, we were unable to identify IRF3 in either PM or BV2 cell lines. Although the IRF3 target gene IFNB was induced in LPS-induced PM cells, it was not induced in BV2 cell lines. Importantly, another IRF3 target gene, CXCL10, was induced in both PM and BV2 cell lines. Our results are similar to those described in a previously published report that showed the induction of IRF3 in dendritic cells and macrophages [47]. Because no IRF3 activation was observed in either type of microglia, the mechanism by which the production of CXCL10 is induced remains obscure. Interestingly, it has been demonstrated that JAK/STAT pathway is a critical player in the regulation of CXCL10 expression in virus- and cytokine-stimulated astrocytes [48]. In addition, other reports also showed that MAP kinase cascades prominently regulate CXCL10 gene expression in microglial cells [49]. Therefore, it seems likely that the LPS-induced induction of CXCL10 transcription depends on JAK/STAT or MAP kinase pathways rather than IRF3 transcriptional pathways in microglial cells. This is an exciting area that we are keenly pursuing further. Importantly, we identified IRF2 and IRF5 as significantly up-regulated only in PM (Fig. 5b, c). In addition, we observed that IRF1, IRF7, and IRF9 were more highly up-regulated in PM than in BV2 cell lines (Fig. 5a, c). Previously, it was demonstrated that IRF, IRF5, and IRF7 may be master regulators that contribute to IRGs [37, 50, 51]. Thus, it would be interesting to explore whether IRG-inducting mechanisms that do not rely on IRF3 exist in cells other than microglia or macrophages. It is therefore likely that IRF1, IRF2, IRF5, IRF7, and IRF9 do not substitute for IRF3 in PM in response to LPS stimulation. A detailed TF target analysis will be required to determine the mechanism by which IRGs are induced in PM cells.

Using this array, we also identified several TFs, including NF-kB, STAT, KLF3, BATF, JUNB, and NFXL1, that have roles in microglial activation that are not well-described (Fig. 5a, b). More importantly, we identified several TFs, including KLF3, BATF, BATF3, FOXP4, NFXL1, and STAT5A, that were uniquely altered in PM (Fig. 5b), suggesting that these TFs might be important regulators in the selective inflammatory gene expression that occurs in these cells. Additionally, our RNA-seq analysis revealed that these and other TFs were more highly expressed in PM than in BV2 cell lines. STAT proteins are critical mediators of immunity to pathogens that are involved in inflammatory diseases [36]. STAT1 and STAT2 are important regulatory factors in the IFN signaling pathways, and they are also essential components of cellular antiviral responses. However, STAT6, KLF9, KLF10, KLF16, and IRF2BP1 were unaffected, suggesting that the induction of TFs in microglial cells is highly selective. To further identify the conserved TF-binding motifs, we performed TF motif analysis using PM cells. Between the two ranges that were available in Pscan that were closest to our region of interest (−950 to 50 and −1000 to 0), the −950- to +50-bp range was selected for the analyses. We found that the promoters of differentially expressed genes were enriched not only for NF-kB transcription factors but also for IRF1, IRF2, STAT1, STAT2, SP1, and SPI1, as shown in Fig. 5f. These analyses provide the first insights into the TF-binding motifs that may be involved in regulating subsets of specific genes in response to LPS stimulation in PM. Next, we used IPA software to identify the target genes that were directly or indirectly activated by the identified TFs (i.e., IRF1, IRF2, STAT1, and STAT2) in response to 4-h LPS. Importantly, we found that the expression of the majority of the LPS-induced stimulated cytokines/chemokines was directly regulated by the identified TFs (Fig. 5g and Table 2).

Unexpectedly, the results in our dataset show that primary microglia express high levels of several epigenetic regulators. Epigenetic regulation is likely to be one of the major mechanisms used by cells to regulate gene expression in response to environmental stimuli [38]. Recently, we showed that the histone demethylase KDM4A and the DNA methyltransferase DNMT3L were strikingly differentially expressed in LPS-induced BV2 cell lines [20]. Importantly, our RNA-seq data revealed that not only KDM4A and DNMT3L but also NSD3, KDM1A, and the SETDB2 were strikingly differentially expressed in LPS-induced PM (Fig. 6a–d). Recently, Schliehe et al. [39] demonstrated that SETDB2 was induced during infection with influenza virus and that it potentially regulates proinflammatory gene expression in macrophages. The mechanism by which NSD3 and SETDB2 become activated following LPS stimulation remains unknown. Determining how these epigenetic regulators, in combination with modified TFs, can regulate distinct sets of inflammatory genes in microglial cells would be intriguing. We suggest that the role of these epigenetic regulators in neuroinflammatory diseases should now be assessed in animal models involving TLR-specific gene deletion or overexpression. However, other histone methyltransferases and *N*-methyltransferases were not expressed in LPS-induced BV2 cell lines and PM cells.

Another interesting finding is that our RNA-seq analysis identified several important differences in the patterns of genes that were uniquely induced by LPS in PM cells (and not induced by LPS in BV2 cell lines). In particular, this technology allowed us to identify over 40 directly LPS-induced genes in these cells (Fig. 6e–g). The results show that almost all of the genes that were associated with inflammation were significantly up-regulated in response to LPS and that their up-regulation was more potent in PM than in BV2 cell lines. Both the extent of the fold changes and the 40 of genes that were changed were significantly increased in PM compared to BV2 cell lines after 2- and 4-h LPS stimulation. The directly LPS-induced genes that are known to be important during activity-regulated processes in PM include LRR and PYD domain-containing protein 3 (NLRP3), which is involved in inflammasomes in MS [52]; caspase (CASP)1; CASP4, which plays a critical role in the processing and secretion of proinflammatory molecules [53]; and PELI1, which is highly expressed in microglia and plays a key role in microglial activation during EAE induction [54]. In this study, we examined BV2 cell lines and PM cells isolated from 3-day-old ICR mice as a model of inflammation studies. Previously, other reports demonstrated that rodent microglia appear to assume similar activation states to human microglia [55, 56]. However the chemotaxis, phagocytosis, transcription regulation, and immunological characteristics have been shown differential expression in adult versus neonatal microglia [14, 57]. Supporting this, previous studies have also demonstrated that microglia derived from neonatal, young, and aged animals are likely to have a unique molecular expression pattern in their responses to activation due to developmental state as well as cell senescence that hinders the cell’s ability to sense changes in their environment [58]. Nevertheless, in the presence of activation states, further studies are warranted to determine the unique transcriptomic signature in different microglial phenotypes as well as the mechanisms by which these LPS-induced genes are expressed and their roles in neuroinflammatory disorders involving microglia.

Microglial cell activation in neuroinflammation is thought to be critically complex. It has been generally thought that microglia can broadly exist in two different states, referred to as the “classically activated,” proinflammatory M1 phenotype and “alternatively activated,” reparative M2 phenotypes [59]. M1 activity can be evoked by LPS, TNF-α, and IFN-γ; induces the production of proinflammatory cytokines, oxidative metabolites, and proteases; and is expected to act as neurotoxic cells, while M2 activation is induced by the stimulation of IL4, IL10, IL13, and TGF-β and may be involved in wound repair and remodeling as well as the production of anti-inflammatory cytokines [60, 61]. Previous studies have shown that adding IL4 before LPS or simultaneous addition of LPS and IL4 significantly dampened the proinflammatory cytokines compared with LPS alone in rat PM cells [62, 63]. These reports are supported by recent results showing that IFN-γ- plus TNF-α-exposed rat PM cells had significantly increased inflammatory mediators as well as receptors/enzymes related to phagocytosis and ROS production but not by IL4 [64]. Similarly, other laboratories provide evidence that in the presence of IFN-γ, but not to IL4 or IL10, BV2 cell lines had significantly increased TNF-α, iNOS, and NO levels and drastically increased expression of NADPH oxidase (NOX2), which plays a critical role in traumatic brain injury (TBI) [65]. Interestingly, Ghosh et al. recently demonstrated that combination of cyclic adenosine monophosphate (cyclic AMP) and IL4, but neither alone, showed ameliorated production of proinflammatory cytokines (TNF-α and IP-10) as well as ROS production in the BV2 cell lines and PM cells [66]. Moreover, there have been data indicating that β-amyloid (Aβ) induces the expression of proinflammatory cytokines in microglia cells, which could lead to AD [67, 68], and IL4 treatment of rat PM cells enhanced uptake and degradation of Aβ [69]. Nevertheless, under classically or alternatively activated conditions, further studies are warranted to determine the unique transcriptomic signature between BV2 cell lines and PM cells.

Overall, our RNA-seq data provide novel insight into the transcriptional differences between cell lines and PM. It is abundantly clear that commonly used microglial BV2 cell lines do not express the same molecular signature as PM after LPS stimulation. The identification of a definitive quantitative PM transcriptome may be useful in determining the features that account for microglia neuroinflammatory functions and their neurotoxic versus neuroprotective properties. In the future, this model can be extended to include data from other high-dimensional surveys, such as microRNA, ChIP-seq, and proteomics, to provide further insight into the global gene regulation processes that occur in LPS-induced microglia.

## Conclusions

In summary, using RNA-seq, we compared the transcriptome changes of microglia BV2 cell lines and PM following stimulation of LPS. Our study demonstrates that PM reacted stronger to LPS and that therefore, a much larger number of transcripts, including different immunoregulatory (cytokines, chemokines, IRGs, etc.) genes, different TFs, and epigenetic regulators, as well as undetected transcripts, were altered in PM than in BV2 cell lines. Furthermore, we identified several specific gene families involved in immune responses that were uniquely altered in LPS-treated PM cells. Our findings thus provide new insights into microglial biology and probably will require more focus on PM activation studies.

## Abbreviations

Aβ, β-amyloid; BP, biological process; CASP, caspase; CNS, central nervous system; CSF, cerebrospinal fluid; cyclic AMP, cyclic adenosine monophosphate; DAVID, Database for Annotation, Visualization and Integrated Discovery; DEG, differentially expressed gene; DMEM, Dulbecco’s modified Eagle’s medium; EAE, experimental autoimmune encephalomyelitis; FBP, fructose-1,6-bisphosphatase; FBS, fetal bovine serum; GAPDH, glyceraldehyde-3-phosphate dehydrogenase; GO, gene ontology; GRO, growth-regulated oncogenes; HDAC, histone deacetylase genes; HMOX, heme oxygenase; IACUC, Institutional Animal Care and Use Committee; IFIT, IFN-induced protein with tetratricopeptide; IFN, interferon; IL, interleukin; IPA, Ingenuity Pathway Analysis; IRG, IFN-regulated gene; KDM, lysine (K)-specific demethylase; KLF, Kruppel-like factor; LPS, lipopolysaccharide; MCP, monocyte chemoattractant proteins; MCSF, macrophage colony-stimulating factor; MF, molecular function; miRNA, microRNA; MS, multiple sclerosis; NLRP, LRR and PYD domain-containing protein; NO, nitric oxide; NOX, NADPH oxidase; PCA, principal component analysis; PGE_2_, prostaglandin E2; PM, primary microglia; RNA-seq, RNA sequencing; ROS, reactive oxygen species; SDF, stromal-derived factor; TBI, traumatic brain injury; TF, transcription factor; TGF-ß, transforming growth factor beta; TLR, toll-like receptor; TNF-α, tumor necrosis factor-alpha

## Additional files

Additional file 1: Figure S1. Morphology and quantification of CD11b-positive microglial cells. (A) Photomicrographs are representative images of BV2 cell lines and PM at 4 h with and without (control) treatment with LPS. Images are at ×20 magnification. (B) Microglial purity is accomplished using flow cytometry. 95.83 and 92.32 % of cells obtained were BV2 cell lines and PM microglia, respectively, as quantified by CD11b. The labeled cells are represented by the purple- and red-shaded populations. (TIF 21196 kb) Additional file 2: Figure S2. PCA analysis of LPS-treated and untreated BV2 cell lines and PM cells. The first principal component (PC1) separates the untreated and LPS-treated samples, while the second principal component (PC2) separates the biological replicates of the same population in BV2 cell lines and PM. (TIF 9666 kb) Additional file 3: Figure S3. Identification of unique and shared genes after 2-h LPS stimulation in BV2 cell lines and PM. Pie chart displaying the number of up or down-regulated genes at 2-h LPS stimulation in BV2 cell lines and PM (upper panel). The area of overlap indicates the number of unique or shared up-regulated genes after 2 h of LPS stimulation in BV2 cell lines and PM (lower panel). (TIF 15155 kb) Additional file 4: Figure S4. Functional annotations of 4-h LPS-inducible down-regulated genes in PM. Gene ontology analysis of functional annotations (biological processes) associated with the top 150 LPS-inducible down-regulated genes at 4 h in PM compared to the control. (TIF 5365 kb) Additional file 5: Table S1. Unique up-regulated genes after 2 and 4 h of LPS stimulation in BV2 cell lines. (DOC 98304 kb) Additional file 6: Figure S5. UCSC Browser images (left panel) representing normalized RNA-seq read densities and transcript abundance (right panel) (in read count) was evaluated in HMOX1 gene using RNA-seq in 2- and 4-h LPS-induced BV2 cell lines and PM cells. (TIF 16998 kb)
