# Supplementary figures and images for: Transcriptome sequencing reveals that LPS-triggered transcriptional responses in established microglia BV2 cell lines are poorly representative of primary microglia
Source: J Neuroinflammation. 2016 Jul 11;13:182. doi: 10.1186/s12974-016-0644-1 (PMC4940985; doi:10.1186/s12974-016-0644-1)

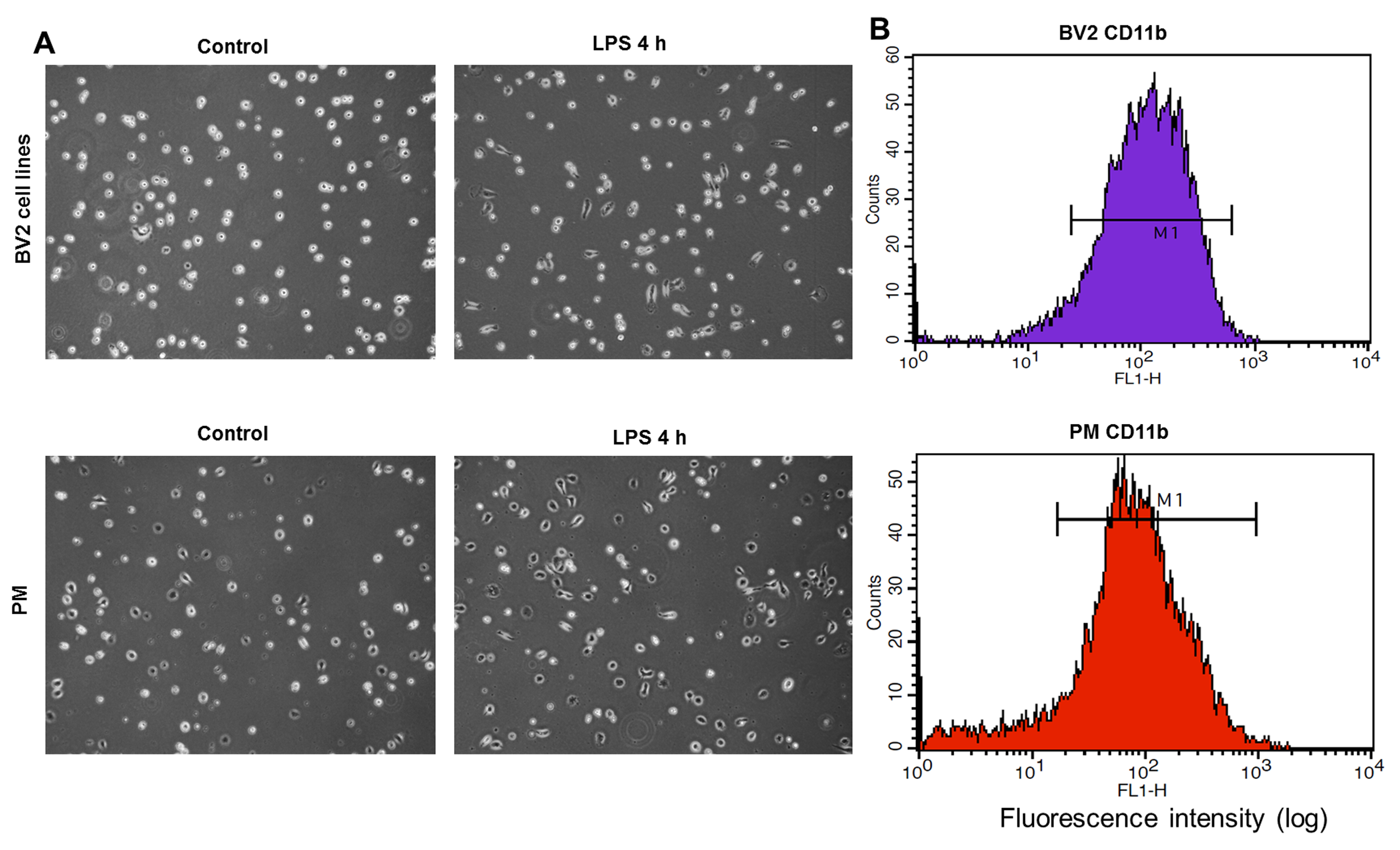

Supplement: Additional file 1: Figure S1. — Morphology and quantification of CD11b-positive microglial cells. (A) Photomicrographs are representative images of BV2 cell lines and PM at 4 h with and without (control) treatment with LPS. Images are at ×20 magnification. (B) Microglial purity is accomplished using flow cytometry. 95.83 and 92.32 % of cells obtained were BV2 cell lines and PM microglia, respectively, as quantified by CD11b. The labeled cells are represented by the purple- and red-shaded populations. (TIF 21196 kb) [file 12974_2016_644_MOESM1_ESM.tif]

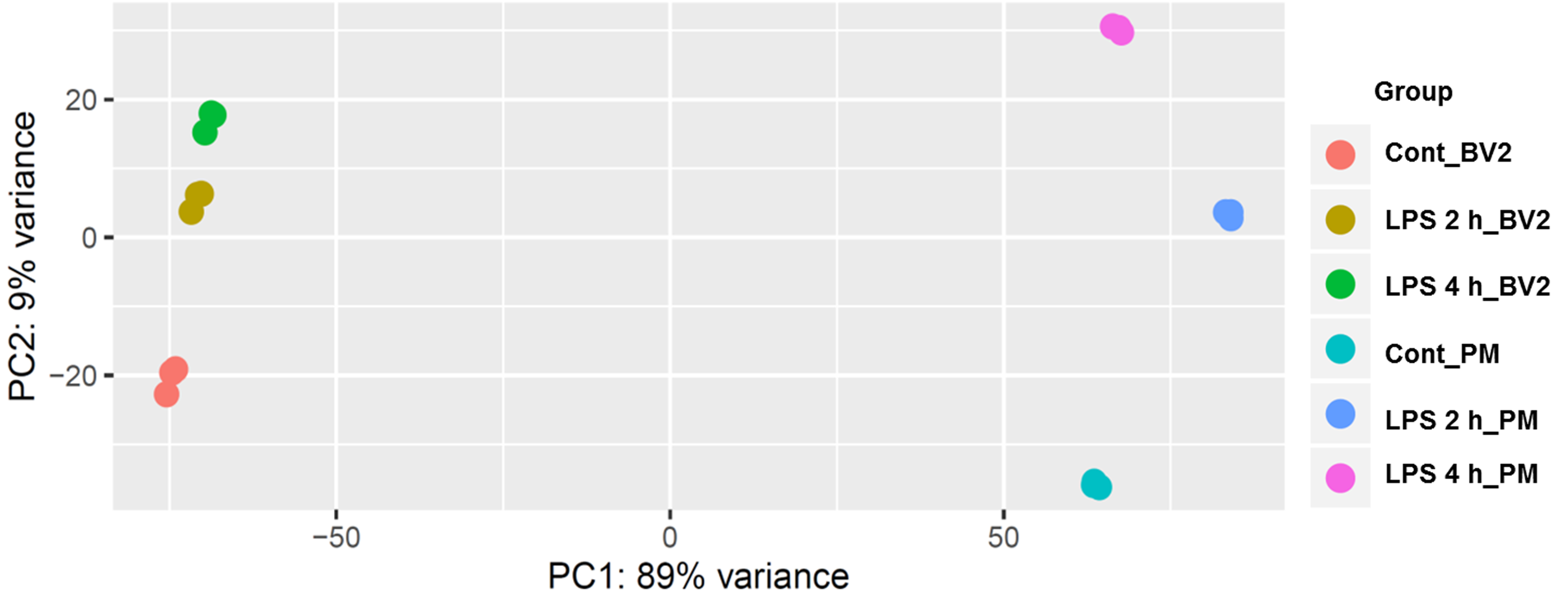

Supplement: Additional file 2: Figure S2. — PCA analysis of LPS-treated and untreated BV2 cell lines and PM cells. The first principal component (PC1) separates the untreated and LPS-treated samples, while the second principal component (PC2) separates the biological replicates of the same population in BV2 cell lines and PM. (TIF 9666 kb) [file 12974_2016_644_MOESM2_ESM.tif]

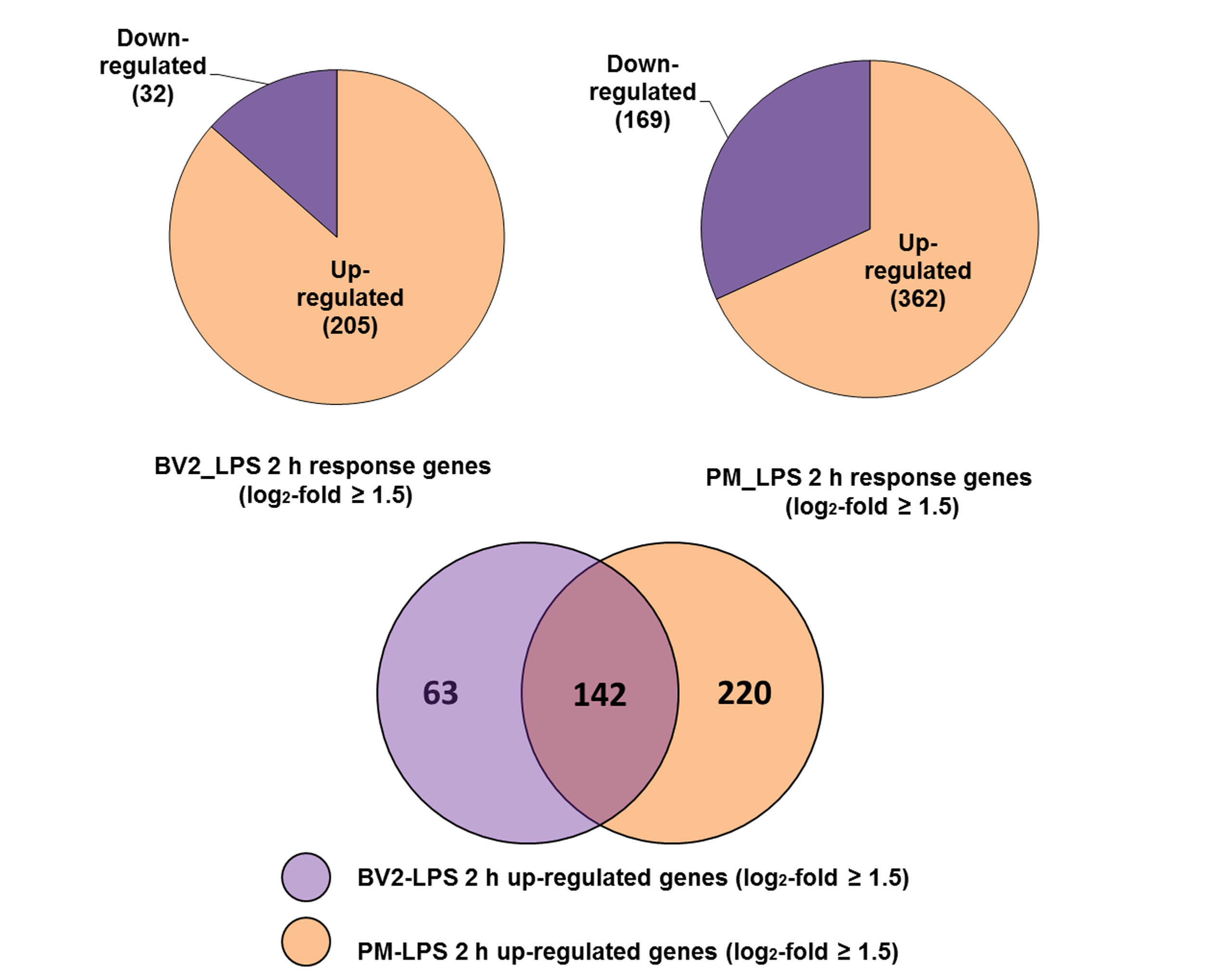

Supplement: Additional file 3: Figure S3. — Identification of unique and shared genes after 2-h LPS stimulation in BV2 cell lines and PM. Pie chart displaying the number of up or down-regulated genes at 2-h LPS stimulation in BV2 cell lines and PM (upper panel). The area of overlap indicates the number of unique or shared up-regulated genes after 2 h of LPS stimulation in BV2 cell lines and PM (lower panel). (TIF 15155 kb) [file 12974_2016_644_MOESM3_ESM.tif]

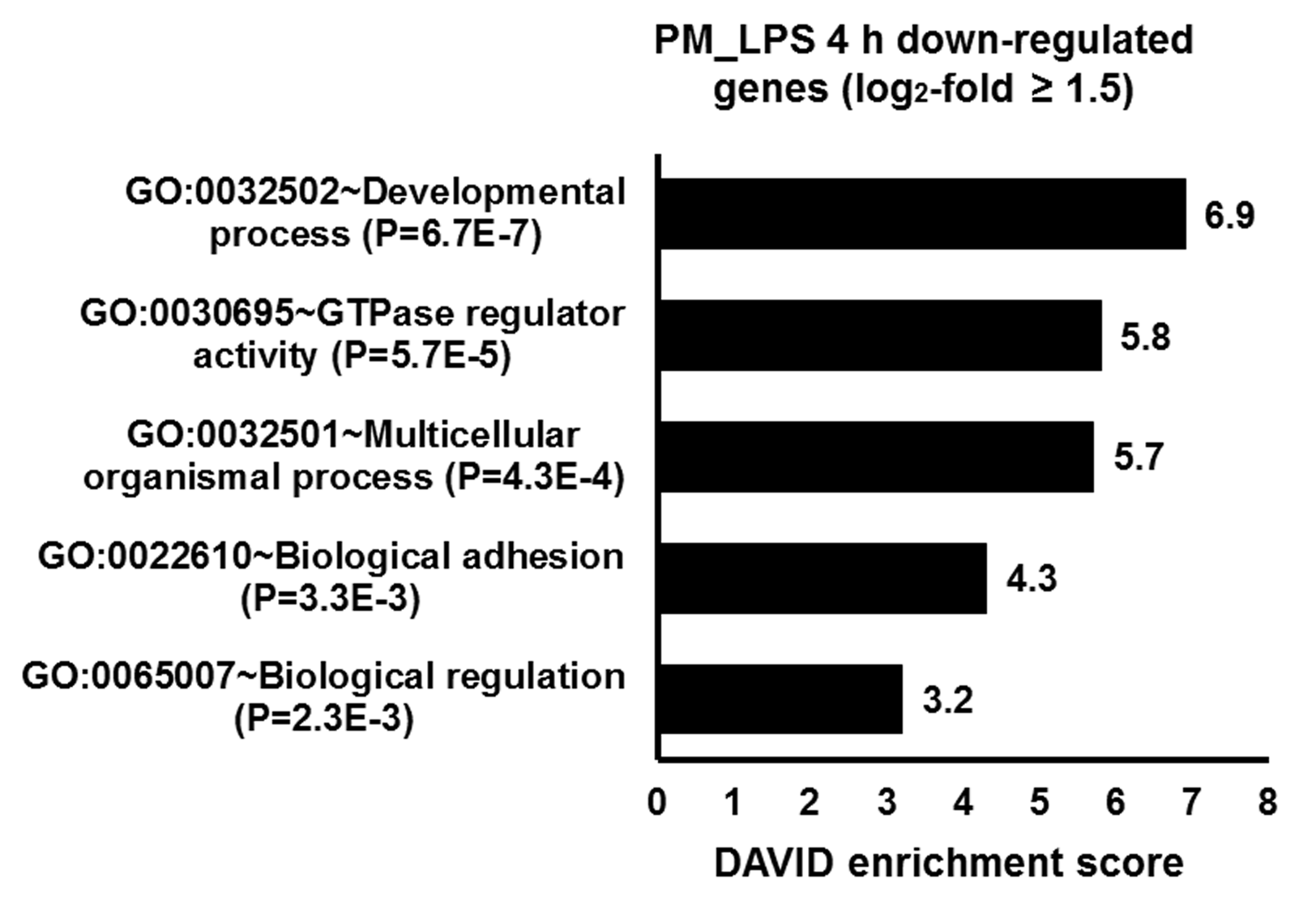

Supplement: Additional file 4: Figure S4. — Functional annotations of 4-h LPS-inducible down-regulated genes in PM. Gene ontology analysis of functional annotations (biological processes) associated with the top 150 LPS-inducible down-regulated genes at 4 h in PM compared to the control. (TIF 5365 kb) [file 12974_2016_644_MOESM4_ESM.tif]

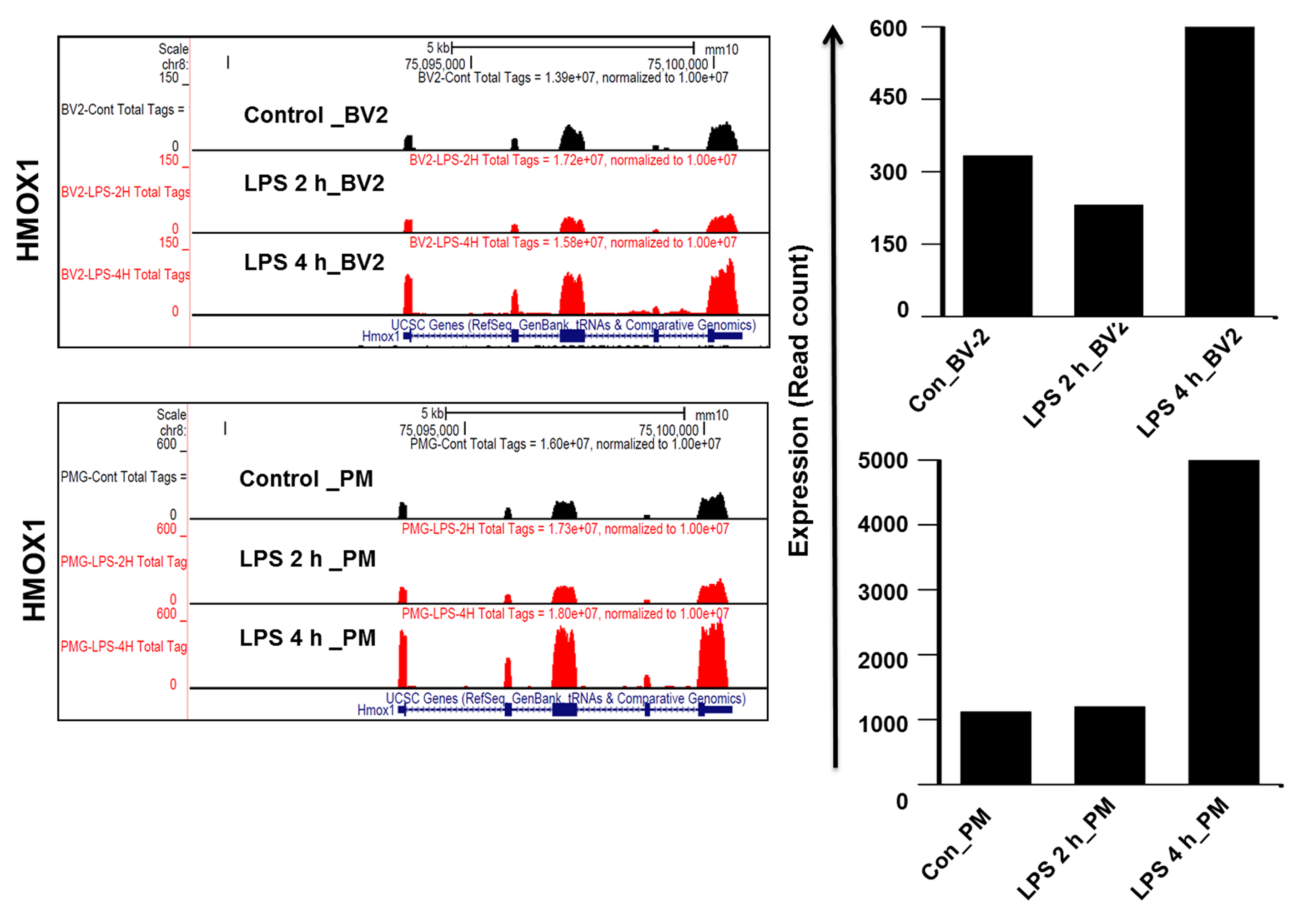

Supplement: Additional file 6: Figure S5. — UCSC Browser images (left panel) representing normalized RNA-seq read densities and transcript abundance (right panel) (in read count) was evaluated in HMOX1 gene using RNA-seq in 2- and 4-h LPS-induced BV2 cell lines and PM cells. (TIF 16998 kb) [file 12974_2016_644_MOESM6_ESM.tif]
