# Supplementary material for: Transcriptome sequencing reveals that LPS-triggered transcriptional responses in established microglia BV2 cell lines are poorly representative of primary microglia
Source: J Neuroinflammation. 2016 Jul 11;13:182. doi: 10.1186/s12974-016-0644-1 (PMC4940985; doi:10.1186/s12974-016-0644-1)
Supplement: Additional file 5: Table S1. — Unique up-regulated genes after 2 and 4 h of LPS stimulation in BV2 cell lines. (DOC 98304 kb) [file 12974_2016_644_MOESM5_ESM.doc]

**Table S1. Unique up-regulated genes after 2 h of LPS stimulation in BV2 cell lines**

| **Gene symbol** | **log_2_ (fold_change)** | ***p*_value** |
| --- | --- | --- |
| SERPINE1 | 5.47 | 1.00E-67 |
| TREML4 | 3.62 | 1.24E-15 |
| CSF3 | 3.52 | 1.06E-14 |
| SELE | 3.17 | 7.98E-12 |
| CCL22 | 3.05 | 5.57E-11 |
| LCN2 | 2.80 | 1.47E-11 |
| HDC | 2.54 | 2.43E-08 |
| FAS | 2.51 | 6.37E-08 |
| GADD45A | 2.38 | 1.66E-07 |
| OSGIN2 | 2.32 | 1.08E-10 |
| MFSD7A | 2.30 | 6.54E-07 |
| KDM4A | 2.29 | 1.39E-22 |
| AMPD3 | 2.28 | 5.71E-14 |
| MAB21L3 | 2.22 | 2.20E-06 |
| ADAMTS4 | 2.21 | 8.07E-07 |
| DUSP8 | 2.20 | 3.61E-06 |
| RALGDS | 2.19 | 2.96E-10 |
| PRDM1 | 2.19 | 1.63E-07 |
| RHOQ | 2.09 | 1.27E-05 |
| BCL2A1B | 2.09 | 1.22E-07 |
| BCL3 | 2.08 | 4.57E-06 |
| ST3GAL1 | 2.05 | 1.69E-16 |
| DCBLD2 | 2.04 | 7.31E-12 |
| THBS1 | 2.04 | 1.21E-08 |
| ARHGEF3 | 2.01 | 5.38E-09 |
| HIVEP2 | 2.01 | 1.72E-07 |
| PHLDA1 | 1.99 | 2.81E-05 |
| MARCKS | 1.98 | 1.28E-08 |
| RSBN1 | 1.98 | 1.11E-09 |
| OSBP2 | 1.95 | 1.89E-05 |
| BCAR1 | 1.93 | 9.43E-06 |
| NR4A3 | 1.86 | 9.26E-05 |
| NR4A1 | 1.83 | 4.37E-06 |
| PPAP2B | 1.83 | 7.70E-06 |
| TMEM171 | 1.82 | 4.65E-05 |
| CDK5R1 | 1.82 | 2.68E-05 |
| LRRC8D | 1.81 | 7.02E-08 |
| TNFRSF9 | 1.80 | 2.55E-06 |
| FRMD6 | 1.78 | 1.17E-05 |
| N4BP1 | 1.69 | 7.44E-13 |
| SLC31A2 | 1.69 | 1.13E-08 |
| METRNL | 1.68 | 6.86E-08 |
| LAMC2 | 1.67 | 5.39E-05 |
| FAM129A | 1.66 | 0.000509901 |
| ZFP697 | 1.65 | 1.63E-05 |
| CSF1 | 1.64 | 7.53E-13 |
| MICALL2 | 1.61 | 6.54E-06 |
| ABCA1 | 1.61 | 4.98E-06 |
| RHOU | 1.61 | 2.55E-05 |
| AGO3 | 1.60 | 9.36E-06 |
| CREM | 1.60 | 1.79E-05 |
| PLAT | 1.60 | 0.000715735 |
| RAB20 | 1.60 | 1.09E-05 |
| NFE2L2 | 1.58 | 4.90E-15 |
| PTPRJ | 1.57 | 2.96E-09 |
| ZFP558 | 1.56 | 0.001020999 |
| RHOB | 1.55 | 2.52E-07 |
| TARM1 | 1.54 | 0.000870956 |
| TRIM36 | 1.53 | 1.26E-05 |
| XYLT2 | 1.51 | 1.11E-06 |

**Unique up-regulated genes after 4 h of LPS stimulation in BV2 cell lines**

| **Gene Symbol** | **log_2_ (fold_change)** | ***p*_value** |
| --- | --- | --- |
| TREML4 | 4.50 | 7.55E-19 |
| APOL9A | 4.05 | 1.13E-16 |
| RILPL1 | 3.30 | 4.28E-11 |
| SERPINE1 | 3.24 | 3.18E-25 |
| TARM1 | 3.04 | 1.36E-10 |
| MFAP3L | 2.95 | 5.64E-14 |
| APOL9B | 2.71 | 4.73E-08 |
| OSBP2 | 2.58 | 1.46E-07 |
| TNFRSF9 | 2.57 | 1.26E-11 |
| LRRC16A | 2.47 | 7.16E-07 |
| OSM | 2.33 | 3.48E-11 |
| NOTCH1 | 2.32 | 1.14E-11 |
| TRIM12A | 2.27 | 5.18E-08 |
| PPAP2B | 2.11 | 9.78E-07 |
| RHOQ | 1.98 | 5.14E-09 |
| LRRC25 | 1.96 | 3.80E-10 |
| FAM49A | 1.96 | 6.74E-08 |
| LRRC8D | 1.86 | 8.85E-09 |
| DUSP1 | 1.85 | 2.66E-05 |
| DUSP5 | 1.84 | 3.79E-06 |
| SERPINB6B | 1.75 | 9.88E-08 |
| NEURL3 | 1.75 | 7.88E-12 |
| DAAM1 | 1.75 | 1.31E-06 |
| PPAP2A | 1.73 | 4.99E-10 |
| KDM4A | 1.67 | 6.60E-13 |
| RAI14 | 1.67 | 7.87E-12 |
| GCNT2 | 1.66 | 9.48E-08 |
| RHOU | 1.62 | 4.76E-05 |
| THBS1 | 1.60 | 3.40E-05 |
| MDM2 | 1.57 | 1.24E-05 |
| RNF149 | 1.57 | 1.99E-08 |
| ZFP719 | 1.52 | 0.000150827 |
| ODC1 | 1.51 | 1.04E-19 |
